# Spatiotemporal trends in cattle lungworm disease (*Dictyocaulus viviparus*) in Great Britain from 1975 to 2014

**DOI:** 10.1136/vr.105509

**Published:** 2020-06-25

**Authors:** Catherine McCarthy, Jan van Dijk

**Affiliations:** 1 Department of Epidemiology and Population Health, University of Liverpool, Chester, Cheshire, UK; 2 Department of Epidemiology and Surveillance, Animal Health Trust, Newmarket, Suffolk, UK

**Keywords:** Dictyocaulus (lungworm), cattle, ostertagia, parasitology, incidence, disease surveillance

## Abstract

**Background:**

Clinical disease caused by the bovine lungworm (*Dictyocaulus viviparus*) causes significant welfare and economic problems for the livestock industry. Anecdotal reports suggest that the number of clinical cases has increased, particularly in Northern England and Scotland. However, these spatiotemporal changes have not been quantified and the current impact that the disease is having across Great Britain remains unclear.

**Methods:**

Here, we report a retrospective analysis of the spatial distribution, seasonality and age of lungworm cases reported by the Veterinary Investigation Diagnosis Analysis database from 1975 to 2014.

**Results:**

A sharp overall increase in the dictyocaulosis diagnostic rate (DR, cases / 1000 submissions) was observed, with, for example, median 2010–2014 DR 3.5 times as high as 1980–1984 DR. Such increases were most pronounced for Scotland, which became the region with the highest proportion of cases by 2009. Cases were increasingly diagnosed during the winter months (December–February).

**Conclusion:**

The apparent spatiotemporal changes in lungworm epidemiology pose new challenges to cattle farmers in Great Britain. Farmers and veterinarians need to remain vigilant for this disease and consider it as a possible cause for milk production losses at any time of the year. Awareness levels may have to be raised particularly in northern England and Scotland.

## Introduction

Bovine parasitic bronchitis (‘Husk’), caused by the lungworm, *Dictyocaulus viviparus*, has long been recognised as a cause of morbidity and mortality in cattle. Gross pathological changes include atelectasis, vesicular and subpleural emphysema and consolidation of apical and cardiac lung lobes.^1 2^ Microscopically, pathology is associated with bronchial plugs, mixed infiltration with necrotic foci and secondary bacterial invasion.^1 3^ Tachypnoea, dyspnoea, weight loss and reduced milk yield are all common features of the disease.^4^ *D. viviparus* infections are associated with reactivation of previous infectious bovine rhinotracheitis (Bovine Herpes Virus 1) infections^5^ which can complicate the clinical signs, often leading to profuse nasal discharge. The respiratory distress caused by lungworm is a serious animal welfare problem.

As well as having welfare implications, lungworm is a disease of significant economic importance. Estimates of milk production losses vary depending on both farm-specific factors and infection levels. Patent infections have been associated with an average daily milk yield loss of 1.62 kg/cow/day.^6^ Clinical lungworm outbreaks are even more costly. Conservative estimates based on two clinical outbreaks in the Netherlands ranged from £11 029 to £17 473 (£100–£116/adult cow in the herd), with a significantly higher acute milk production loss of 4 kg/cow/day.^7^

During the 1950s, lungworm was considered to be one of the key diseases most damaging to the cattle industry.^8^ In 1959, a live vaccine, containing irradiated larvae, was launched and rapidly became a cornerstone in the control of the disease.^4^ The 1980s saw the advent of long-acting anthelmintic treatments and an ever-increasing reliance upon these to control gastrointestinal and respiratory nematodes affecting cattle.^8^ Despite these control methods, and the fact that *D. viviparus* anthelmintic resistance (AR) has not been reported in the UK, the number of lungworm cases recorded in the Veterinary Investigation Diagnosis Analysis (VIDA) database increased significantly during the 1990s.^4 9^ Moreover, the number of cases in older cattle has increased, whereas up to the early 1990s it had been regarded as a disease of young stock.^9 10^ Quantifying any epidemiological changes is the first step towards understanding possible causes behind any such changes. However, no robust analyses have been performed on spatially and temporally explicit data on lungworm cases in Great Britain.

The VIDA surveillance database collates reports from regional veterinary surveillance laboratories to monitor the incidence rates of exotic and endemic diseases of cattle, sheep, pigs and poultry in Great Britain. This is a passive surveillance system allowing voluntary submission of samples, or carcases for post-mortem examination, by farmers through their veterinarians. Lungworm disease is not notifiable and so reports may be subjected to reporting bias. While potential sources of bias and confounding have to be acknowledged, a key strength of the VIDA database is the length of time during which data have been collected. Moreover, the diagnostic protocols for helminth parasites have remained identical for decades. The VIDA database therefore functions as a useful resource for detecting long-term temporal and spatial trends.

The aim of the present study is to perform a robust statistical analysis of the spatiotemporal trends in lungworm disease of cattle from 1975 to 2014. Temporal trends in annual and seasonal disease abundance, regional disease trends and distribution of disease over different age classes of animals are analysed, with a view to facilitating local vigilance and disease control planning. Furthermore, this study aims to provide baseline data against which future changes in spatiotemporal disease abundance can be measured. Finally, we explore hypotheses for the drivers behind the observed trends which could be explored in further research.

## Materials and methods

The VIDA database records every submission made to the regional laboratories of the Animal and Plant Health Agencies Veterinary Investigation centres in England and Wales and the Scottish Agricultural College (SAC) in Scotland. Data were collected on both the total number of submissions and the number of cases of dictyocaulosis in the database from 1975 to 2014. To facilitate the generation of hypothesis on drivers of recorded changes in lungworm epidemiology, dictyocaulosis incidence trends were compared to those in ostertagiosis and unspecified parasitic gastroenteritis (PGE). During 2014, regional laboratories contributing to VIDA were dismantled with the number of diagnostic laboratories was reduced from 14 to 6.^11^ As such, a dramatic change in regional disease recording is likely to have introduced reporting bias, data on the most recent years (2014–2018) were discarded.

The diagnostic criteria became centrally defined in 1999 but continued, as for pre-1999, to be based on the judgement of experts in veterinary pathology and disease surveillance. The techniques used to identify parasites within carcases or dung have not significantly changed since 1975. The only change to lungworm diagnostic protocols was the introduction of an ELISA in 1991 which detected antibodies to the male adult worms (sperm antigen).^12–14^ However, seropositivity, or eosinophilia alone, wase not sufficient evidence to classify a positive case (table 1). ‘Ostertagiosis’ was diagnosed from post-mortem samples in late winter or early spring and typically refers to the syndrome known as type 2 ostertagiosis (disease caused by the synchronous re-emergence of previously hypobiotic *Ostertagia ostertagi* larvae). Unspecified PGE in cattle is usually caused by *O. ostertagi* (type 1 disease) and *Cooperia* spp. (primarily *Cooperia oncophora*) in animals in their first or second grazing season at pasture, and diagnosed ante-mortem, for example, through faecal egg testing.

**Table 1:** Case definitions of dictyocaulosis, ostertagiosis and unspecified PGE in cattle recorded in the Veterinary Investigation Diagnosis Analysis database

Parasitic disease	Case definition
Dictyocaulosis	Relevant clinical history and/or gross pathology and either (a) demonstration of *Dictyocaulus viviparus* in the bronchial tree, (b) detection of first stage larvae (L1) in the faeces or (c) histopathology
Ostertagiosis	Relevant clinical history and significant numbers (approximately 20 000 or more) of *Ostertagia ostertagi* adults/larvae in the abomasum
Unspecified PGE	Relevant clinical history and/or gross pathology and/or histopathology and either (a) detection of worms in gastrointestinal tract, (b) FEC (usually >500 egg per gram (EPG) in individual faecal egg count (FEC) examinations) or (c) larval culture/identification

FEC, fecal egg count; PGE, parasitic gastroenteritis.

All statistical analyses were performed using the R statistical software.^15^ The number of lungworm cases diagnosed on each date (month and year) and age of cattle was compared to the total number of cattle samples submitted to the network of laboratories (for any diagnostic reason) in each month and age range to calculate a diagnostic rate (DR; lungworm cases per 1000 submissions). Data on the total number of submissions were only available from 1979 onwards and so DRs were calculated from 1979. Spearman’s rank correlation was used to test for significance in changes of the DR from 1979 to 2014. To assess more detailed DR changes over time, years were divided into seven lots of 5-year blocks (1980–1984, 1985–1989, 1990–1994, 1995–1999, 2000–2004, 2005–2009, 2010–2014). The Kruskal–Wallis test was used to assess whether there were any significant differences between the time blocks and Mann–Whitney U post-hoc tests were applied to identify significance between individual time blocks, as has previously been described.^16^ To understand the changes in dictyocaulosis in the context of other common parasitic diseases, annual data were also assessed for ostertagiosis and unspecified PGE.

Data were categorised according to the following regional levels: southwest, southeast, east, midwest, north England, Scotland and Wales (see appendix). To assess the relative frequency of lungworm disease in each region over time, and to allow for annual variations in lungworm intensity, the proportion of the total GB cases which was recorded from each region was calculated for all years. Regional data were only available from 1999. Changes in the proportions from 1999 to 2014 for each region were assessed using Spearman’s rank correlation test.

To compare inter-year variations in disease intensity, the median and IQR of the number of lungworm cases recorded per month in each region was calculated for both 1980–1990 and 2000–2010. In addition, the seasonal distribution of cases was assessed by calculating the number of cases recorded in each month as a proportion of the total number of cases recorded over the year. This was calculated for individual years (1975–2014) and for each region. Trends were assessed using the Spearman’s rank correlation test.

The proportion of cases diagnosed in young stock under 2-year old (either first season grazing calves, second-year grazing, non-lactating heifers or male second season stock) out of the total number of submissions in young stock was calculated over each decade (1980–1989, 1990–1999 and 2000–2009) and compared to the proportion in adult cattle (over 2-years old) using the χ^2^ test. The OR and Wald’s CIs of case being under 2-years old was calculated for each decade.

## Results

**Figure 1:**
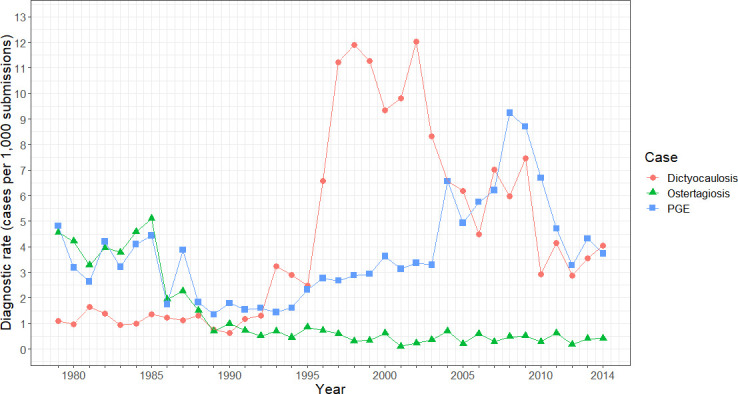
Diagnostic rate (cases per 1000 cattle submissions) of dictyocaulosis (red circles), ostertagiosis (green triangles) and PGE (blue squares) in Great Britain from 1979 to 2014. Adapted from C. McLeonard and J. van Dijk, in practice, 2017, 39. PGE, parasitic gastroenteritis.

A total of 7616 lungworm cases were diagnosed from 1975 to 2014. There was an overall significant increase in the dictyocaulosis DR between 1979 and 2014 (r_s_=0.65, p<0.001), with DRs approximately increasing fourfold over this timeframe (figure 1). DRs peaked in 2002 with 12.03 cases/1000 submissions. There were significant differences between the 5-year timeframes (H_(6)_=27.3, p<0.001). Each 5-year block between 1980 and 1994 had a significantly lower DR than blocks between 1995 and 2009 (table 2). There was a significant decrease in DRs between 2000–2004 (median 9.35 cases/1000 submissions) and both 2005–2009 (median 6.18/1000 submissions, U=23, p=0.03) and 2010–2014 (median 3.54/1000 submissions, *U*=25, p=0.01).

**Table 2:** Median diagnostic rates (cases / 1000 submissions) of dictyocaulosis, ostertagiosis and parasitic gastroenteritis (PGE) recorded in the Veterinary Investigation Diagnosis Analysis (VIDA) surveillance database

Five-year timeframe	Median dictyocaulosis rates (cases/1000 submissions)	Median ostertagiosis rates (cases/1000 submissions)	Median PGE rates (cases/1000 submissions)
1980–1984	1.00^a, b, c^	3.95^a, b, c, d, e^	3.21^a, b^
1985–1989	1.22^d, e, f^	1.94^ f, g, h, i^	1.83^c^
1990–1994	1.31^ g, h, i^	0.70^a, j^	1.60^a, d, e, f, g^
1995–1999	11.22^a, d, g^	0.60^b, f^	2.77^d, h, I, j^
2000–2004	9.35^b, e, h, j, k^	0.35^ c, g^	3.37^e, h^
2005–2009	6.18^ c, f, i, j^	0.48^d, h^	6.21^b, c, f, i^
2010–2014	3.54^k^	0.40^e, i, j^	4.32^ g, j^

Letters (a–j) refer to significant differences between pairs for each parasitic disease according to the Mann–Whitney U test.

The DR of ostertagiosis significantly decreased between 1979 and 2014 (r_s_=−0.81, p<0.001). There were significant differences between the 5-year blocks in ostertagiosis DRs (H_(6)_ =22.9, p<0.001) with each 5-year block between 1980 and 1989 having a significantly higher rate of each 5-year block between 1995 and 2014 (table 2). Rates of PGE significantly increased from 1979 to 2014 (r_s_=0.46, p<0.001) with significant differences between each 5-year block (H_(6)_=24.3, p<0.001). The 5-year blocks between 1990 and 1999 all had significantly lower DRs than between 2000 and 2014.

**Figure 2:**
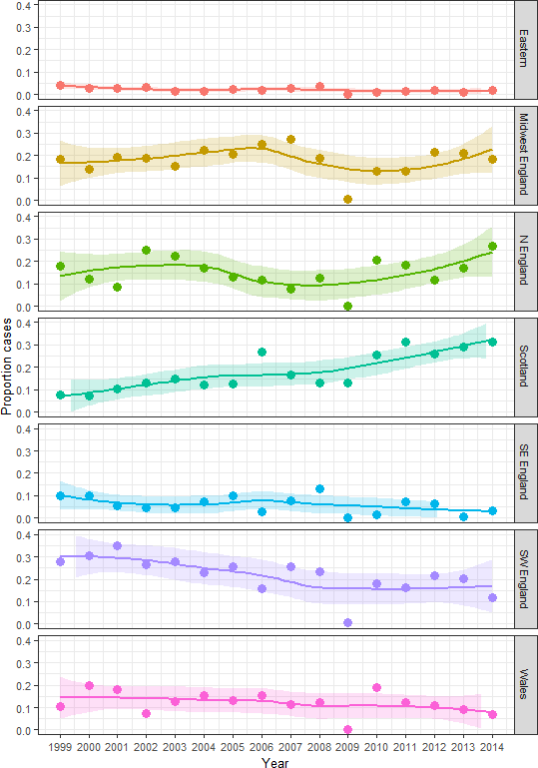
Lungworm cases recorded in each region by the Veterinary Investigation Diagnosis Analysis database from 1999 to 2014 as a proportion of the total number of cases recorded in that year. Shaded regions show Loess smoothing.

The proportion of total dictyocaulosis cases diagnosed from southwest and east England approximately halved between 1999 and 2014 (southwest England: 0.28 of total cases in 1999 to 0.12 of total cases in 2014, r_s_=−0.82, p<0.001 and east England: 0.04 of total cases in 1999 to 0.02 of total cases in 2014, r_s_=−0.62, p=0.01) (figure 2). The proportion of cases diagnosed in Scotland significantly and strongly increased from 1999 to 2014 (r_s_=0.86, p<0.001) with proportions in 2014 (0.31 of total cases) being around 4.5 times higher than in 1999 (0.08 of total cases). From 2009 onwards, Scotland has recorded the highest number of cases than any region across Great Britain. DRs from southeast, midwest, northern England and Wales did not change significantly between 1999 and 2014.

**Figure 3:**
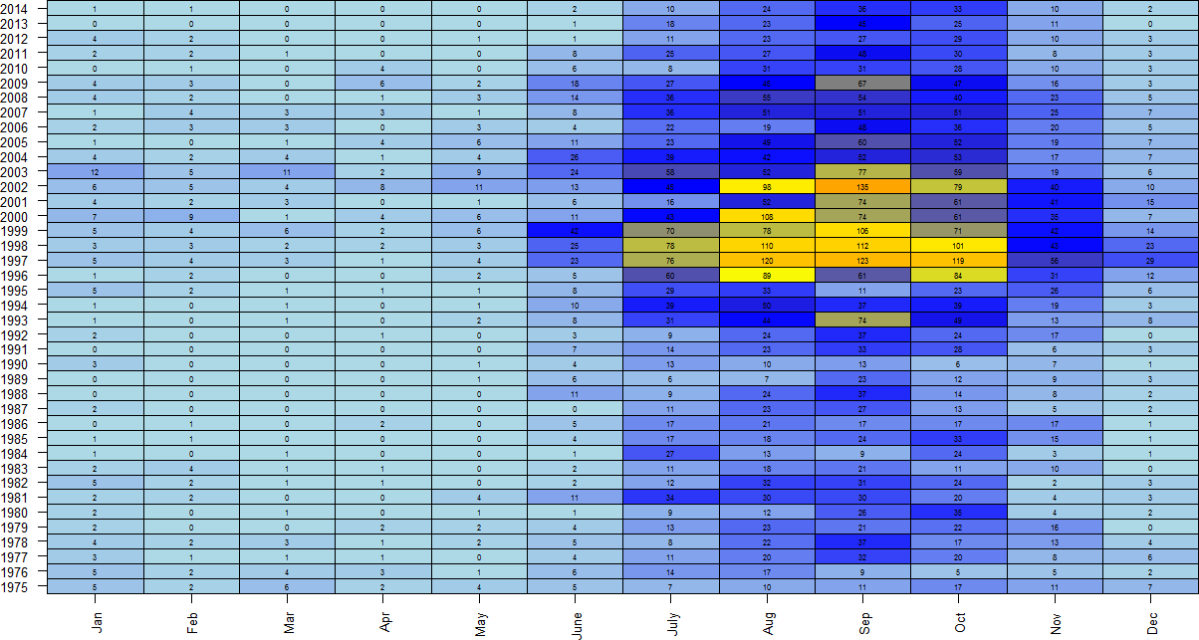
Monthly distribution of lungworm cases recorded on the Veterinary Investigation Diagnosis Analysis database from any region of great Britain 1975 to 2014 (rows). Heatmap colours refer to number of cases from light blue (fewest cases) to dark orange (most cases). Numbers within cells refer to the number of cases diagnosed per month and year.

**Figure 4:**
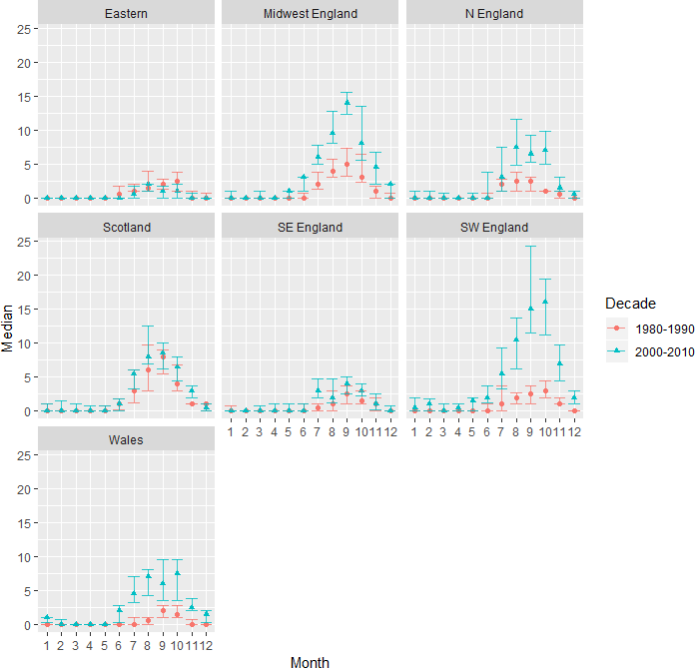
Median (with 25% and 75% quantiles) number of lungworm cases recorded in each month and each region from 1980 to 1990 (red line) and 2000–2010 (blue line).

The majority of lungworm cases from 1975 to 2014 (0.78 total cases) were recorded between July and October (figure 3). The variation between years was more extreme from 2000 to 2010 than from 1980 to 1990, with July to November in southwest England becoming particularly unpredictable (figure 4). There was a change in seasonality between 1975 and 2014, with an increasing number of cases reported during the winter, specifically December to February in southwest England; November to January in Wales; and March in northern England (table 3). Cases were recorded in June in midwest England and Wales; and October in northern England. There was a decrease in the number of cases recorded in January and June in Scotland.

**Table 3:** Changes in seasonality of dictyocaulosis cases from 1975 to 2014 as recorded by the Veterinary Investigation Diagnosis Analysis database

Region	January	February	March	April	May	June	July	August	September	October	November	December
Southwest England	0.37 (0.02)	0.43 (0.01)	-	-	-	-	-	-	-	-	-	0.32 (0.05)
Southeast England	-	-	-	-	-	-	-	-	-	-	-	-
East England	-	-	-	-	-	-	-	-	-	-	-	-
Midwest England	-	-	-	-	-	0.31 (0.05)	-	-	-	-	-	-
Northern England	-	-	0.34 (0.04)	-	-	-	-	-	-	0.55 (<0.001)	-	-
Scotland	−0.37 (0.02)	-	-	-	-	−0.43 (0.01)	-	-	-	-	-	-
Wales	0.35 (0.03)	-	-	-	-	0.31 (0.05)	-	-	-	-	0.39 (0.01)	0.36 (0.02)

Note that only significant results (p≤0.05) are shown.

Numbers refer to the Spearman’s rank correlation test results with p values in parenthesis.

**Figure 5:**
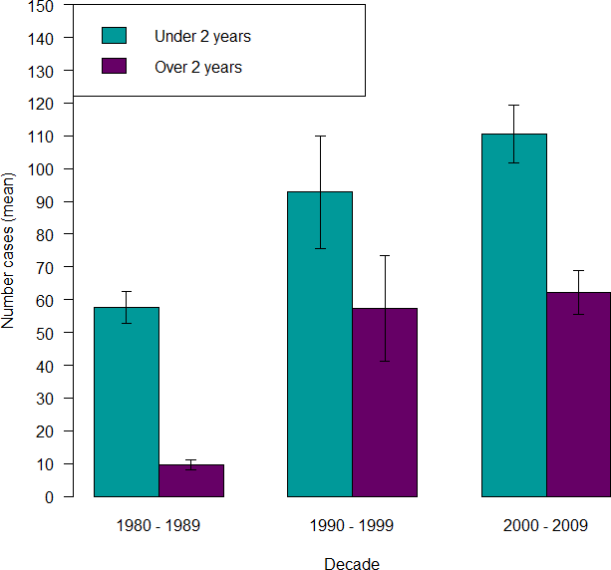
Average age of lungworm cases (mean and standard error of mean, vertical bars) recorded in the Veterinary Investigation Diagnosis Analysis database by decade.

During the 1980s, the odds of a lungworm case occurring in cattle under 2 years of age was 5.22 times higher than for adult cattle (95% CI 4.20 to 6.48, χ_2_=280.3, p<0.001) (figure 5). This was also significant for the 1990s (3.28 higher odds, 95% CI 2.96 to 3.64, χ_2_=560.8, p<0.001) and 2000s (3.37 higher odds, 95% CI 3.05 to 3.71, χ_2_=657.8, p<0.001).

## Discussion

As recorded in the VIDA database, the overall annual DR of clinical dictyocaulosis in Great Britain rose significantly between 1980 and 2014, with a particularly large increase between 1995 and 2000. Median DRs significantly decreased again from 2000 to 2004 and 2005–2014. However, they remained significantly higher than the levels pre-1995, with current lungworm incidence approximately four times as high as in the 1980s. It should be remembered that the VIDA database is a passive surveillance system and so is likely to involve a degree of under-reporting. Moreover, under-reporting rates may show temporal and spatial changes and should not assume to be static across the four decades or in each region. Arguably in areas or years where the disease has become more prevalent, there may be an increased reluctance to send cases for diagnosis (as farmers and vets become more likely to assume the diagnosis). Therefore, the decrease in reported cases since 2000 may reflect a degree of reporting bias.

There were sharply contrasting, significant, differences in regional trends across Great Britain. The proportion of cases diagnosed in southwest and eastern England decreased significantly since 1999. In contrast, the proportion of cases recorded in Scotland increased dramatically over the same timeframe and by 2009, for the first time, Scotland became the region where the largest proportion of cases was recorded. The increase in cases in northern England and Scotland from 2009 onwards agrees with numerous reports from Scottish farmers, vets and the SAC.^17^ Important differences in regional epidemiology were also recorded with respect to the between-year variance in cases. In Scotland, although the number of outbreaks is increasing, it appears that the seasonality and total number of cases thus far, follows a predictable pattern. In contrast, in southwest England, the overall number of cases is in decline but the potential for unpredictably high, or aseasonal, outbreaks is still present. If no cases are seen for a few years, this tends to give a false sense of security, potentially leading to a lack of preparedness from both vets and farmers.

Moreover, it appears that changes in seasonality of the parasite are also starting to occur. More cases are starting to be diagnosed during the winter, particularly December to February in southwest England and November to January in Wales. Even in the comparatively cooler northern England, the dictyocaulosis season is expanding into March. The highest risk periods for lungworm (typically coinciding with the late summer/early autumn months) appear to be starting earlier in some regions (June in midwest England and Wales), and ending later in others (October in northern England). The available dataset did not provide details on farm management practices for affected cases. We therefore could not examine factors such as whether cases were from housed or grazing cattle, beef or dairy animals or anthelmintic use on farm. Further work should investigate this emerging trend, particularly in winter cases, to understand factors driving these changes.

Previous reports have described the increasing number of outbreaks observed in adult cattle.^9 10 18^ The present paper provides further evidence that disease incidence is increasing in cattle in their third and consecutive grazing seasons. The concurrent changes recorded (eg, increased overall abundance, south-to-north shifts in regional importance, regional differences in between-year variability, expanding seasonality and more frequent recording of disease in adult cows) pose a challenge to the formulation of a single hypothesis potentially explaining all phenomena; it is likely that more than one driver is at play. In the field of gastrointestinal nematodes, changes in epidemiology are often assumed to be at least partly driven by increasing levels of AR. However, although anecdotal field reports are suggestive of its existence, to our knowledge, AR has not been formally documented in *D. viviparus* in Great Britain. Moreover, the recorded seasonal and regional shifts cannot be explained by AR.

The increased overall number of cases, and more cases being seen in adult cattle, has been ascribed to the increased reliance on macrocyclic lactones (MLs) for worm control, preventing the build-up of long-lasting immunity in young cattle and disrupting reinfection needed to boost immunity in adult herds.^19 20^ However, there is some evidence to suggest that calves treated with long-acting anthelmintic boluses may have a degree of immunity acquired in the grazing season during which the anthelmintics were applied.^21–23^ Whether infection while covered by MLs will result in reliable immunity is likely to depend on the infection levels at pasture. DRs of ostertagiosis declined from 1980 to 1999. The key reason for this is likely to be the introduction of MLs, which were advocated for use at housing since the 1990s to treat inhibited *O. ostertagi* larvae. Although this suggests that there has been a change in anthelmintic prescribing habits, it is unlikely to explain the regional differences in lungworm epidemiology described here.

There are several limitations in using a passive surveillance system to make inferences on disease incidence rates and the findings observed should be considered in light of the socioeconomic changes of the time. It should not be assumed that management practices (and therefore lungworm risk) would remain static across this 40-year timeframe. Changes in cattle breeds and genetics, industrialisation of particularly the dairy industry with predominance of single breed cattle, more intensive grassland management and decline of autumn, block calving practices may all influence farm management practices. The increasing need for high-yielding animals may have altered pasture use and driven an increasing use of anthelmintics, with subsequent decreased opportunities to develop natural immunity to *D. viviparus*. The increasing cases from 1995 to 2003 may therefore be partially explained by changing farm practices. Note that the bifurcated peak, with lower incidence in 2000 and 2001 is likely to reflect the foot-and-mouth disease epidemic of 2001. Licensing of eprinomectin as a novel, nil-milk-withhold, avermectin with high efficacy against *D. viviparus* occurred in 1997.^24^ It is possible that this helped to reduce the case incidence during the 2000s. More work is needed to understand the significance that farm management changes have had on endemic disease incidences.

A decline in the use of lungworm vaccine has also been proposed as a risk factor for an increase in lungworm outbreaks. Vaccine sales declined in the UK since the 1980s.^4^ A survey of the UK farming industry in 1996 finding that only three out of 32 herds with clinical lungworm outbreaks used the vaccine and two of the three herds only saw clinical disease in unvaccinated animals.^4^ During the 1990s, only 20%–25% of dairy herds in England and Wales used the vaccine whereas in Scotland, this figure was closer to 50%.^4^ After the bovine spongiform encephalitis epidemic in the UK, the lungworm vaccine was withdrawn from the Netherlands in 1996 which led farmers to adopt other preventative measures such as long-acting anthelmintic treatments.^20^ This withdrawal of the vaccine coincides with the timing of the major peak in lungworm incidence from 1996 to 2003. To understand the impact that declining vaccination use has had on the incidence of lungworm disease, further studies are needed to quantify the current uptake of the vaccine and whether there remains any spatial differences in vaccination use. In particular, qualitative studies are needed to investigate motivations and barriers behind the use of the vaccination.

Although it is possible that the socioeconomic changes have influenced lungworm disease rates, this fails to explain some of the seasonal and regional differences observed in this analysis. The increased trend of PGE reported here has also been recorded for several species of gastrointestinal nematodes of sheep^16^ and *Fasciola hepatica* in cattle and sheep.^25^ It has been demonstrated that such changes can be ascribed to changes in temperature and rainfall.^26 27^ Despite the suppressive effect of anthelmintics on parasite populations, climate-driven increases in parasite abundance are commonly witnessed across helminths affecting ruminants, for example as both the transmission season of parasites and grazing season of hosts expand at the same time. Older scientific papers discuss some of the effects of temperature and moisture on the development and survival of the free-living stages of *D. viviparus*,^28^ but this work has not been repeated using modern, more robust scientific practices. It has been proposed that development of the parasite is relatively independent of temperature^29^ and that outbreaks are normally seen following periods of rainfall. The south-to-north, and seasonal, changes described here may suggest that climate changes have played a role in influencing the severity of disease outbreaks. Further work is needed to confirm or disprove whether climate change has had any effect on the disease incidence.

According to the VIDA database, there has been an increase in the DRs of lungworm disease across Great Britain from 1979 to 2014 with a significant increase in cases located in northern England and Scotland from 2009. There is evidence of a shift in the seasonality of cases which implies that the disease is becoming more unpredictable. In similarity with other parasitic diseases, this could be explained through the early effects of climate change. Farmers and veterinarians must remain vigilant for this disease. It should be considered as a possible cause for milk production losses, with or without respiratory symptoms, at any time of year and not only in late summer and early autumn.
